# Calorimetric Evaluation of Glycyrrhetic Acid (GA)- and Stearyl Glycyrrhetinate (SG)-Loaded Solid Lipid Nanoparticle Interactions with a Model Biomembrane

**DOI:** 10.3390/molecules26164903

**Published:** 2021-08-13

**Authors:** Debora Santonocito, Carmelo Puglia, Cristina Torrisi, Alessandro Giuffrida, Valentina Greco, Francesco Castelli, Maria Grazia Sarpietro

**Affiliations:** 1 Department of Drug and Health Sciences, University of Catania, Viale Andrea Doria 6, 95125 Catania, Italy; debora.santonocito@unict.it (D.S.); cristina.torrisi@phd.unict.it (C.T.); fcastelli@unict.it (F.C.); mg.sarpietro@unict.it (M.G.S.); 2 Department of Chemical Sciences, University of Catania, Viale Andrea Doria 6, 95125 Catania, Italy; alessandro.giuffrida@unict.it (A.G.); vgreco@unict.it (V.G.)

**Keywords:** glycyrrhetic acid, stearyl glycyrrhetinate, solid lipid nanoparticles, calorimetry, biomembrane

## Abstract

Glycyrrhetic acid (GA) and stearyl glycyrrhetinate (SG) are two interesting compounds from *Glycyrrhiza glabra*, showing numerous biological properties widely applied in the pharmaceutical and cosmetic fields. Despite these appreciable benefits, their potential therapeutic properties are strongly compromised due to unfavourable physical-chemical features. The strategy exploited in the present work was to develop solid lipid nanoparticles (SLNs) as carrier systems for GA and SG delivery. Both formulations loaded with GA and SG (GA-SLNs and SG-SLNs, respectively) were prepared by the high shear homogenization coupled to ultrasound (HSH-US) method, and we obtained good technological parameters. DSC was used to evaluate their thermotropic behaviour and ability to act as carriers for GA and SG. The study was conducted by means of a biomembrane model (multilamellar vesicles; MLVs) that simulated the interaction of the carriers with the cellular membrane. Unloaded and loaded SLNs were incubated with the biomembranes, and their interactions were evaluated over time through variations in their calorimetric curves. The results of these studies indicated that GA and SG interact differently with MLVs and SLNs; the interactions of SG-SLNs and GA-SLNs with the biomembrane model showed different variations of the MLVs calorimetric curve and suggest the potential use of SLNs as delivery systems for GA.

## 1. Introduction

**Figure 1:**
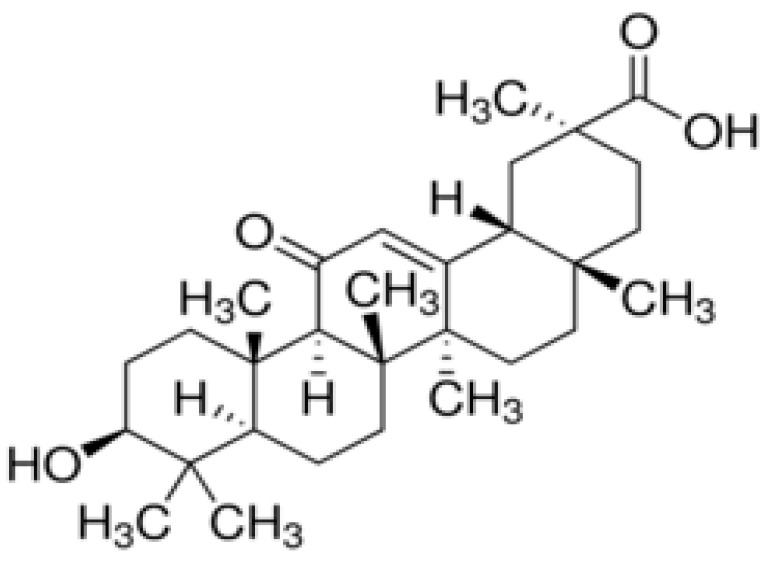
Chemical structure of GA.

Liquorice (*Glycyrrhiza glabra*) is a Leguminosae perennial widely cultivated in the Mediterranean area and central Asia [1]. Liquorice root extract contains interesting compounds used in the pharmacological and cosmetic fields. The major component is 18 β glycyrrhetic acid (GA), a triterpene saponin obtained from the hydrolysis of glycyrrhizin (Figure 1) [2]. GA shows numerous pharmacological activities such as antitumor, antioxidant, antiviral, and anti-inflammatory [3,4,5,6]. The last property is due to the similarity between the structure of the GA and that of cortisone, a steroid hormone with strong anti-inflammatory activity [7]. In the cosmetic field, GA is used as a lenitive and anti-reddening agent [8].

**Figure 2:**
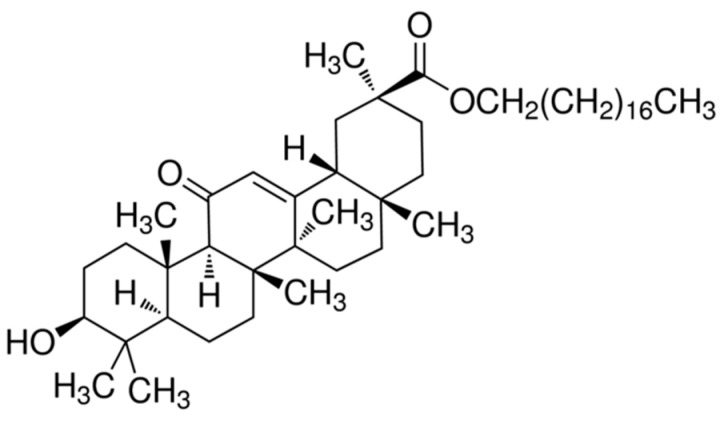
Chemical structure of SG.

Another interesting derivative of liquorice is stearyl glycyrrhetinate (SG), the stearyl ester of 18 β glycyrrhetic acid (Figure 2). SG is a fatty acid widely used in the cosmetic industry due to its skin-soothing and mattifying properties [9], and it is used as an antiviral and anti-inflammatory agent in the pharmaceutical field [4].

Despite these interesting features, GA and SG show some unsuitable physicochemical properties (lipophilic nature, extremely poor water solubility, and low bioavailability) that drastically compromise their therapeutic and/or cosmetic use [3,8,10]. In order to overcome these limits, a nanotechnology approach was developed; specifically, the use of solid lipid nanoparticles (SLNs) can be exploited to deliver and improve the bioavailability of the drugs over time.

SLNs are colloidal carrier systems made of solid biodegradable lipids (Generally Recognized As Safe, GRAS) stabilized by surfactants [11]. SLNs show many advantages such as low cost, high stability, good biocompatibility, and high scale production [12]. Moreover, they are able to incorporate lipophilic and hydrophilic molecules and control their release [11].

Several studies in have demonstrated the importance of a lipid nanoparticle approach in ameliorating the delivery and bioavailability of active pharmaceutical ingredients (APIs) [13,14].

In previous work by our group, palmitoylethanolamide (PEA)-loaded nanostructured lipid carriers (NLCs) were developed to enhance the ocular bioavailability of this drug. Pharmacokinetic studies showed that the retinal levels of PEA were significantly higher in the group treated with a PEA-NLC formulation versus an aqueous suspension of PEA. Therefore, NLCs demonstrated their ability to deliver high levels of lipophilic drugs to the back of the eye after topical ocular administration [13,15].

In another study, SLNs were able to optimize the cutaneous application of idebenone (IDE), an active pharmaceutical ingredient characterized by photo instability and low bioavailability [16]. DSC was used to study the interactions between a lipid matrix and IDE following the transfer kinetics of the APIs from SLNs to a site mimicking a biomembrane.

On the basis of the previous considerations, the aim of the present work was to formulate GA- and SG-loaded SLNs, to investigate the mechanism by which SLNs interact with biological membranes by means of a calorimetric evaluation, and to optimize the delivery of GA and SG. In this regard, a biomembrane model based on liposomal multilamellar vesicles (MLVs) made of dimyristoylphosphatidylcholine (DMPC) was used due to the MLVs’ ability to reproduce the fluidity and mobility of cell membranes. MLVs were also used to study interactions with bioactive agents [17,18].

## 2. Results and Discussion

### 2.1. Characterization of SLNs

GA-SLNs and SG-SLNs at different concentrations (0.25, 0.5, and 0.75% *w/v*) were formulated using Compritol 888ATO as the lipid matrix and Lutrol F68 as the surfactant. We chose Compritol as the lipid matrix due to its high affinity towards GA and SG (data not shown). Furthermore, we decided to investigate different concentrations of GA and SG, although many scientific papers in the literature have found an optimal concentration of 0.5% for both APIs [8,10].

All formulations were prepared by the high shear homogenization coupled to ultrasound (HSH-US) method. As reported in the literature [15], this combination guarantees good technological parameters in terms of mean particle size and polydispersity index; furthermore, Z potential (ZP) values indicated the good stability of the nanoformulations (Table 1). Specifically, the mean particle size remained unchanged and suitable for administration despite the increased drug concentrations.

### 2.2. MLVs and SLNs Analysis

**Figure 3:**
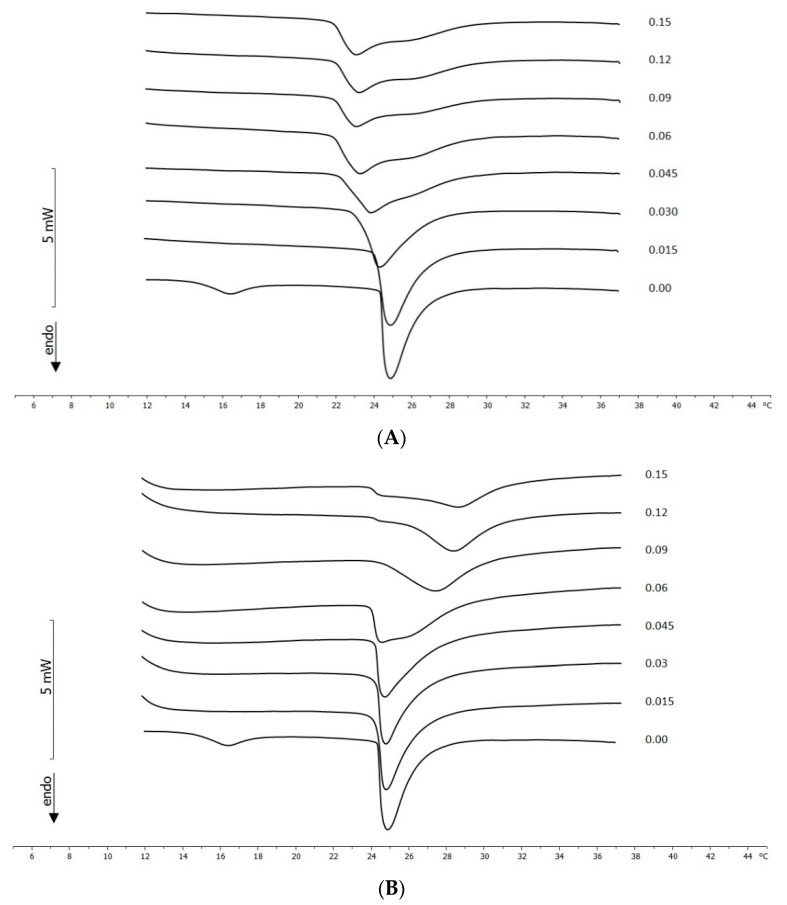
(**A**) Calorimetric curves, in the heating mode, of MLVs prepared with different molar fractions of GA. The labels at the right side of each curve represent the molar fraction of GA. (**B**) Calorimetric curves, in the heating mode, of MLVs prepared with different molar fractions of SG. The labels at the right side of each curve represent the molar fraction of SG.

To evaluate whether GA and SG can interact with DMPC MLVs, we prepared DMPC MLVs containing increasing molar fractions of GA or SG. As reported in the literature [19,20], DMPC is widely used to study the interaction of bioactive compounds and biomembrane models because its transition temperature is about 24 °C; this allows uptake experiments to be carried out at 37 °C, a physiological temperature at which the bilayers are in a disordered liquid crystalline state more favourable for interactions with drug [18]. The eventual interaction of GA and SG with MLVs was studied by comparing the calorimetric curves of DMPC MLVs containing increasing amounts of GA or SG with those of pure DMPC MLVs (Figure 3A,B). The calorimetric curve of the DMPC MLVs were characterized by pre-transition peaks at 16 °C due to the transition from the ordered or gel state to the ripple phase and a main transition peak at about 25 °C due to the transition from the ripple phase to a disordered or liquid crystalline state; any modifications led to interactions with GA or SG.

GA caused variations in the DMPC MLV calorimetric curves; the pre-transition peak disappeared. As the GA molar fraction increased, the main transition peak shifted to lower temperatures and broadened. Starting from the 0.045 molar fraction, a shoulder appeared at a higher temperature than that of the main transition peak. This behaviour is a clear indication of the interaction between GA and DMPC MLVs. In particular, GA exerted a fluidising effect on the MLV bilayers and caused phase separation; in other words, in the structure of the MLVs, phospholipid regions rich in GA and phospholipid regions poor in GA coexisted [21,22].

The calorimetric curves of MLVs prepared with SG differed from the pure DMPC MLVs’ calorimetric curves. The pre-transition peak was absent. The main peak moved up to the 0.045 molar fraction toward a slightly lower temperature, and starting from the 0.06 molar fraction, it moved towards a higher temperatures and broadened. Then, SG interacted with the DMPC MLVs. The effect caused by SG indicated that an interaction between the stearyl moiety of the molecule and the lipophilic part of DMPC occurred. The broadening and shortening of the main peak could have been due to a decrease of cooperativity during the melting among the acyl chain of the phospholipids in the MLVs. The shift of the main transition peak towards a higher temperature suggested the SG interacted with the hydrophobic tails of the phospholipid and seemed to stabilize it; therefore, the temperature of the main transition was higher. This is possible if a system formed by the lipophilic chains of SG and acyl chains of phospholipid are interdigitated, thus forming a complex structure [23].

**Figure 4:**
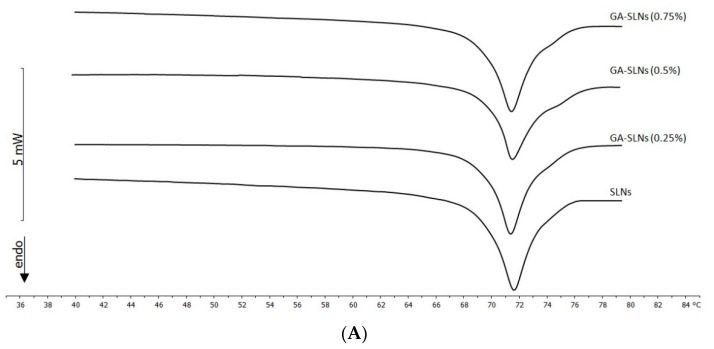

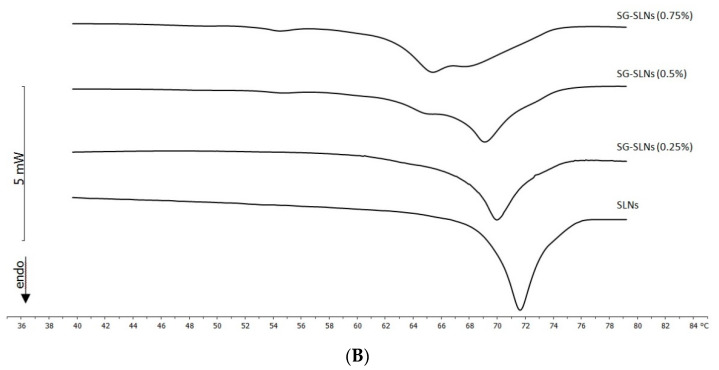
(**A**) Calorimetric curves, in the heating mode, of GA-SLNs. (**B**) Calorimetric curves, in the heating mode, of SG-SLNs.

The calorimetric curves of SLNs, unloaded or containing different amount of GA, are shown in Figure 4A. Unloaded SLNs showed a transition peak centred at 71.59 °C. The melting point of Compritol 888 ATO in SLN form was depressed, showing a slight shift to a lower temperature than that of the bulk lipid (71.74 °C). This melting point depression could have been due to the small particle size, high specific surface area, and presence of a surfactant [24]. The melting enthalpy value of the lipid in the SLN (100 J/gr) formulations decreased compared to that of the bulk lipid (128 J/gr). This lower melting enthalpy value could have been due to the less ordered lattice arrangement of the lipid within nanoparticles compared to the bulk materials [25]. In fact, in the less-ordered structure, the melting of the substance required less energy than the perfect crystalline substance, which needed to overcome lattice force. Lipid nanoparticles seemed to lose part of the crystalline state. The decrease in the melting point and enthalpy value was associated with lattice defects and the formation of amorphous regions in which the molecules could be located.

The presence of GA caused some variation in the calorimetric curves of SLNs. The main peak slightly enlarged, whereas the peak temperature remained almost unchanged. In addition, the shoulder at higher temperature became more evident as the amount of GA increased. This results indicated that GA was almost homogeneously distributed in the SLN structure (Figure 4A).

The effect of SG on the SLNs was quite different. The main peak moved to a lower temperature; at 0.25% of SG, a shoulder appeared at a lower temperature, and its intensity increased when the amount of SG increased. At 0.75% of SG, the shoulder became a well-defined peak. The SG made the SLNs structure more fluid. Additionally, it did not localize in the SLN structure in a homogeneous way; rather, the presence of the two peaks indicated that in the SLN structure, SG-poor regions and SG-rich regions coexisted (Figure 4B).

### 2.3. Kinetic Experiments

#### 2.3.1. Interaction between MLVs and GA/SG

In order to evaluate whether GA and SG could be absorbed by and interact with the MLVs, kinetic experiments were carried out. The solid compounds (GA and SG) were separately put in contact with the MLVs, and variations in the calorimetric curve of MLVs due to the eventual interaction with compound were evaluated as time of contact increased. The melting peak of GA was not visible because it melted at about 294 °C. Regarding MLVs, the pre-transition peak was always present even if shifted to a lower temperature and the main transition peak remained unchanged. This behaviour indicated that GA did not enter the MLV structure (data not shown).

Regarding SG, the peak at 75.5 °C was due to its melting, which did not change for all contact times. The pre-transition and main transition peaks of the MLVs remained unchanged for all contact times, which is a clear sign that the MLVs and SG did not interact with each other (data not shown). Therefore, neither GA nor SG were able to be absorbed and enter the MLV bilayers.

#### 2.3.2. Interaction between MLV and SLNs

**Figure 5:**
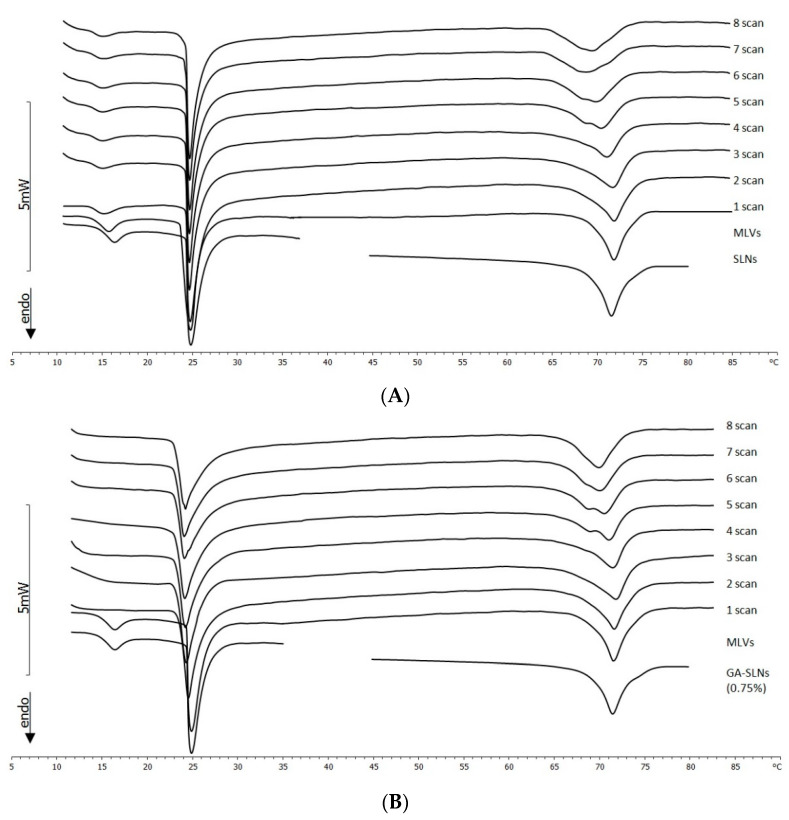

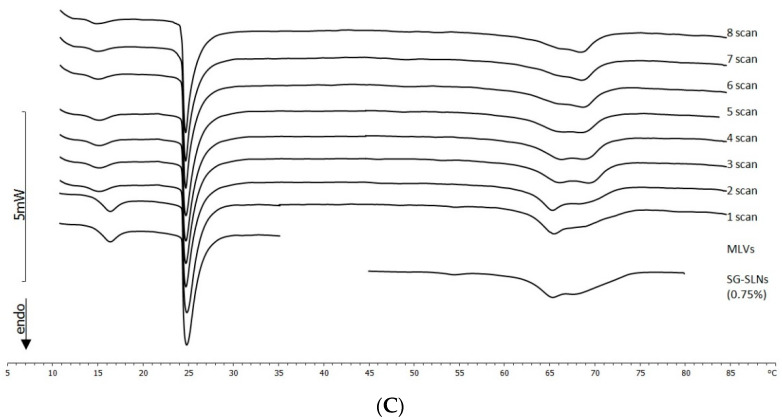
(**A**) Calorimetric curves, in the heating mode, of MLVs put in contact with unloaded SLNs. For comparison, calorimetric curves of the samples put in contact (MLVs and SLNs) are shown. (**B**) Calorimetric curves, in the heating mode, of MLVs put in contact with GA-SLNs (0.75%). For comparison, calorimetric curves of the samples put in contact (MLVs and GA-SLNs (0.75%)) are shown. (**C**) Calorimetric curves, in the heating mode, of MLVs put in contact with SG-SLNs (0.75%). For comparison, calorimetric curves of the samples put in contact (MLVs and SG-SLNs (0.75%)) are shown.

**Figure 6:**
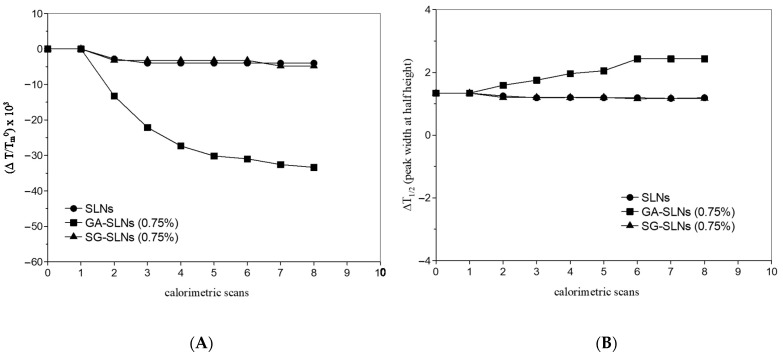
(**A**) Transition temperature variations—made using ΔT/T_m_^0^; ΔT = T_m_–_m_^0^, where T_m_^0^ is the transition temperature of MLVs and T_m_ is the transition temperature of MLVs put in contact with SLNs, GA-SLNs (0.75%), and SG-SLNs (0.75%)—as a function of the calorimetric scans. (**B**) ΔT_1/2_ (peak width at half height; °C) of MLVs put in contact with SLNs, GA-SLNs (0.75%), and SG-SLNs (0.75%) as a function of the calorimetric scans.

To follow the interaction between MLVs and SLNs, the two systems were put in contact in the calorimetric pan and the variation of the calorimetric curves was recorded. To obtain information on the mechanism of the eventual interaction, first, MLVs and unloaded SLNs were put in contact. In the calorimetric curves (Figure 5A), the peaks relative to the MLVs and the peaks related to the SLNs are recognizable. MLVs retained the pre-transition and main transition peaks. The pre-transition peak was slightly changed, whereas the main transition peak retained its shape and intensity. The values related to the transition temperature and the ΔT_1/2_ (Figure 6A,B, respectively) did not show significant variations with respect to the values of the MLVs. Even the enthalpy variation did not vary with respect with the enthalpy variation of the MLVs (data not shown). The SLNs peak varied during the incubation, and in the fifth scan, a shoulder that became more evident in subsequent scans appeared. This is evidence that the MLVs maintained their peaks without deeply interacting with the SLNs. The SLNs did not maintain their structure, as suggested by the appearance of the shoulder. Additionally, they were not able to enter the MLV structure. Pre-transition, rather than being a core property of the phospholipid bilayer (as with the main transition), depends on the surface structure of the membrane and is related to the reorientation of the phospholipid head group and water [26]; therefore, the variation of the MLV pre-transition peak could have been due to the interactions of the SLNs with the DMPC bilayer surface and the consequent perturbation of the periodic ripple organization [27].

The calorimetric curves of MLVs put in contact with GA-SLNs (0.75%) revealed a quite different scenario (Figure 5B). The MLVs lost the pre-transition peak. The main transition peak shifted to a lower temperature and broadened; from the sixth scan, a second signal at a higher temperature appeared. The enthalpy variation remained almost unchanged for all times of contact (data not shown), the transition temperature decreased (Figure 6A) (suggesting the fluidization of the MLVs), and the ΔT_1/2_ increased (Figure 6B). This parameter was used as a measure for the cooperativity of transition, that is the number of phospholipid molecules undergoing simultaneous transition; in particular, ΔT_1/2_ was inversely proportional to cooperativity. Then, in the MLVs, a decrease of the cooperativity of the phospholipid transition occurred. The SLNs peak varied: at the beginning of contact, a single peak was present; then, it split into two peaks that, as the contact time increased, merged into a unique peak. All these signs clearly indicated that MLVs interacted with the SLNs. Because unloaded SLNs did not enter the MLVs, instead just interacting with their surfaces, we can hypothesize that the GA-SLNs (0.75%) interacted with the MLV surface and released GA into the MLVs. Moreover, a comparison of the main transition peaks of the MLVs with the main transition peaks of MLVs prepared with GA revealed great similarities such as the disappearance of the pre-transition peak, the appearance of the shoulder at higher temperature, and the enlargement of the main peak transition; these signs seemed to confirm that a release of GA from SLNs to MLV occurred.

The calorimetric curves of the experiment that put MLVs and SG-SLNs (0.75%) in contact are depicted in Figure 5C. The pre-transition peak of the MLVs was slightly shifted towards a lower temperature. The main transition peak remained unchanged even after a long time of contact. The enthalpy variation remained unchanged (data not shown). The transition temperature (Figure 6A) and ΔT_1/2_ (Figure 6B) underwent slight variations. The signal relative to SG-SLNs changed during contact time: the peak at a lower temperature decreased in favour of the peak at a higher temperature. Therefore, the structure of the SG-SLNs changed during the time. This result suggests that SG-SLNs and MLVs only slightly interacted. In a similar way to unloaded SLNs, SG-SLNs could have interacted with the MLV surface; the strong interactions taking place among SG and SLN molecules did not permit SG to be transferred from SLNs to MLVs. This behaviour was confirmed by the release studies (data not shown); SG was not released from the nanocarriers due to its extremely lipophilic structure (insoluble). On the other hand, GA-SLNs showed poor release due to the less lipophilic nature of GA compared to SG (<0.01 mg/mL) [28].

## 3. Materials and Methods

### 3.1. Materials

GA (18-β-glycyrrhetic acid) was a gift from A.C.E.F. S.p.A. (Piacenza, Italy), while SG (stearyl glycyrrhetinate) was purchased from Maruzen Pharmaceuticals Co., Ltd. (Hiroshima, Japan). Pluronic^®^ F68 (poloxamer 188), was purchased from the BASF Corporation (Florham Park, NJ, USA). Compritol^®^ 888 ATO (glyceryl behenate, tribehenin)—= a mixture of mono-, di-, and triglycerides of behenic acid—was a gift from Gattefossè (Milan, Italy). Dimyristoylphosphatidylcholine (DMPC) was purchased from Genyme (Liestal, Switzerland). All other materials were of analytical grade.

### 3.2. Preparation of SLNs

GA-SLNs and SG-SLNs were prepared by the high shear homogenization coupled to ultrasound (HSH-US) method, as described in [29] with some modifications. Briefly, GA or SG (0.25, 0.5, and 0.75% *w*/*v*) was dissolved in a lipid phase containing Compritol 888 ATO (5.0 g), and the mixture was stirred at 80 °C to obtain a dispersion. The melted lipid phase was dispersed in the hot (80 °C) surfactant solution (Lutrol F68, 1.5% *w*/*v*) by using a high-speed stirrer (Ultra-Turrax T25, IKA-Werke GmbH & Co. Kg, Staufen, Germany). The obtained pre-emulsion was ultrasonicated by a Labsonic 2000 (B. Braun, Melsunen, Germany) for 8 min. The hot dispersion was then cooled by dilution in 100 mL of additional water at 4 °C. Unloaded SLNs were prepared by the same procedure without adding GA or SG.

### 3.3. Characterization of SLNs

Dynamic light scattering (DLS; Zetasizer Nano S90; Malvern Instruments, Malvern, UK) was employed to measure the mean particle size, polydispersity index (PDI), and ZP of SG-SLNs and GA-SLNs using a 90° scattering angle and a solid-state laser (4.5 mW, 670 nm). Samples were measured after dilution (1:10, SLNs/double distilled water) at 25 °C. Each value was measured at least in triplicate.

### 3.4. DMPC Multilamellar Vesicles (MLVs) Preparation

DMPC MLVs were obtained as follows: DMPC was dissolved in chloroform:methanol (1:1, *v*/*v*). Then, the solvents were evaporated under nitrogen flow at 37 °C. The obtained phospholipid film was freeze-dried over night to eliminate eventual solvent traces, and 50 mM of a Tris buffer solution at pH 7.4 was added to the film to reach 0.1229 mmoles DMPC/mL. The phospholipid dispersion was kept at 37 °C for 1 min, vortexed for 1 min three times, and then kept at 37 °C for 60 min.

### 3.5. DMPC/GA MLVs and DMPC/SG MLVs Preparation

DMPC/GA MLVs and DMPC/SG MLVs were obtained as follows: DMPC, GA, and SG were separately dissolved in chloroform:methanol (1:1, *v*/*v*). Aliquots of a DMPC solution (containing 0.010325 mmoles) were delivered in glass vials, and aliquots of the GA or SG solution were added to reach defined molar fractions of compounds with respect to DMPC (0.00, 0.015, 0.03, 0.045, 0.06, 0.09, 0.12, and 0.15). The solvents were evaporated under nitrogen flow at 37 °C. The obtained phospholipid films were freeze-dried over night to eliminate eventual solvent traces, and 50 mM of a Tris buffer solution at pH 7.4 was added to the films to reach 0.0614 mmoles DMPC/mL. The phospholipid dispersions were kept at 37 °C for 1 min. vortexed for 1 min three times, and then kept at 37 °C for 60 min.

### 3.6. Differential Scanning Calorimetry (DSC)

DSC studies were carried out with a Mettler Toledo STAR^e^ system equipped with a DSC-822^e^ calorimetric cell and the Mettler TA-STAR^e^ software. We employed 160 μL aluminium pans, which were sealed after filling. The reference pan contained a Tris buffer solution. The sensitivity was automatically chosen as the maximum possible by the calorimetric system that was calibrated, in temperature and enthalpy changes, by using indium, stearic acid, and cyclohexane following the procedure of the Mettler TA STAR^e^ software.

### 3.7. MLVs and SLNs Analysis

MLVs (120 μL; 0.007375 mmoles of DMPC) were put into the calorimetric pan and subjected to DSC analysis as follows: they underwent (1) a heating scan from 5 to 37 °C at a rate of 2 °C/min and then (2) a cooling scan from 37 to 5 °C at a rate of 4 °C/min; each scan was conducted at least three times to check the reproducibility of the results. SLNs (120 μL) were put into the calorimetric pan and subjected to DSC analysis as follows: they underwent (1) a heating scan from 25 to 80 °C at a rate of 2 °C/min and then (2) a cooling scan from 37 to 5 °C at a rate of 4 °C/min; each scan was conducted at least three times to check the reproducibility of the results.

### 3.8. Kinetic Experiments

#### 3.8.1. Interaction between MLVs and GA/SG

GA and SG were separately weight in the calorimetric pan, and 120 μL of a MLV sample was added. Then, the pan was hermetically sealed, and the interaction was monitored for DSC analysis as follows: the pan underwent (i) a heating scan at a rate of 2 °C/min between 5 and 80 °C (ii) a cooling scan at a rate of 4 °C/min between 80 and 37 °C, (iii) an isothermal period (1 h) at 37 °C to allow the SLNs to interact with and permeate the phospholipid bilayers of MLVs that (at the chosen temperatures above the lipid transition temperature) were in a disordered state, and (iv) a cooling scan between 37 and 5 °C at a rate of 4 °C/min to bring the phospholipid bilayers back to an ordered state before restarting the heating program. This procedure was run at least eight times to follow eventual variations in the transition temperature of the DMPC MLVs due to the time-dependent interactions. Each experiment was repeated three times.

#### 3.8.2. Interaction between MLVs and SLNs

Sixty microlitres of MLVs (0.1229 mmoles DMPC/mL) were placed in the calorimetric pan, followed by the addition of 60 μL of SLNs; the pan was hermetically sealed, and the interaction was monitored before submitting the sample to DSC analysis as described above.

## 4. Conclusions

Glycyrrhetic acid (GA) and stearyl glycyrrhetinate (SG) are two interesting compounds from *Glycyrrhiza glabra* that are used in the pharmacological and cosmetic fields. Though both compounds express high pharmacological potential, they are often not therapeutically used due to their poor water solubility. This obstacle can be overcome by encapsulating these compounds into SLNs. GA-SLNs and SG-SLNs were prepared by the high shear homogenization coupled to ultrasound (HSH-US) method, and we obtained good technological parameters. The ability of SLNs to delivery lipophilic drugs, such as GA and SG, was evaluated by using the DSC and multilamellar vesicles of DMPC, which was chosen as a biomembrane model. The DSC technique allowed us to study the interactions between the lipid matrix and bioactive compounds, as well as how they diffuse from the lipid-based nanocarrier to the site mimicking a biological membrane. Calorimetric results indicated that GA and SG interact differently with MLVs and SLNs, suggesting their potential use as delivery systems for these compounds.

## Figures and Tables

**Table 1:** Z-Ave, PDI, and ZP values for unloaded SLNs, GA-SLNs, and SG-SLNs at different concentrations (0.25, 0.5, and 0.75% *w/v*).

Formulation	Z-Ave [nm ± SD]	PDI [-] ± SD	ZP [mV ± SD]
Unloaded SLNs	157.3 ± 0.25	0.361 ± 0.35	−27.9 ± 0.14
GA-SLNs (0.25%)	210.0 ± 0.09	0.286 ± 0.22	−26.2 ± 0.10
GA-SLNs (0.5%)	159.2 ± 0.23	0.336 ± 0.25	−40.3 ± 0.09
GA-SLNs (0.75%)	215.1 ± 0.05	0.276 ± 0.21	−41.8 ± 0.50
SG-SLNs (0.25%)	174.2 ± 0.02	0.249 ± 0.40	−26.3 ± 0.25
SG-SLNs (0.5%)	198.8 ± 0.28	0.247 ± 0.03	−36.1 ± 0.20
SG-SLNs (0.75%)	160.0 ± 0.07	0.189 ± 0.34	−39.1 ± 0.06

## Data Availability

The data used to support the findings of this study are available from the corresponding author upon request.
